# Genome-wide linkage scan for maximum and length-dependent knee muscle strength in young men: significant evidence for linkage at chromosome 14q24.3

**DOI:** 10.1136/jmg.2007.055277

**Published:** 2008-01-03

**Authors:** G De Mars, A Windelinckx, W Huygens, M W Peeters, G P Beunen, J Aerssens, R Vlietinck, M A I Thomis

**Affiliations:** 1 Department of Biomedical Kinesiology, Research Center for Exercise and Health, Faculty of Kinesiology and Rehabilitation Sciences, Katholieke Universiteit Leuven, Leuven, Belgium; 2 Department of Translational Medical Research, TIBOTEC BVBA, Mechelen, Belgium; 3 Department of Human Genetics, Faculty of Medicine, Katholieke Universiteit Leuven, Leuven, Belgium

## Abstract

**Background:**

Maintenance of high muscular fitness is positively related to bone health, functionality in daily life and increasing insulin sensitivity, and negatively related to falls and fractures, morbidity and mortality. Heritability of muscle strength phenotypes ranges between 31% and 95%, but little is known about the identity of the genes underlying this complex trait. As a first attempt, this genome-wide linkage study aimed to identify chromosomal regions linked to muscle and bone cross-sectional area, isometric knee flexion and extension torque, and torque–length relationship for knee flexors and extensors.

**Methods:**

In total, 283 informative male siblings (17–36 years old), belonging to 105 families, were used to conduct a genome-wide SNP-based multipoint linkage analysis.

**Results:**

The strongest evidence for linkage was found for the torque–length relationship of the knee flexors at 14q24.3 (LOD  = 4.09; p<10^−5^). Suggestive evidence for linkage was found at 14q32.2 (LOD  = 3.00; P = 0.005) for muscle and bone cross-sectional area, at 2p24.2 (LOD  = 2.57; p = 0.01) for isometric knee torque at 30° flexion, at 1q21.3, 2p23.3 and 18q11.2 (LOD  = 2.33, 2.69 and 2.21; p<10^−4^ for all) for the torque–length relationship of the knee extensors and at 18p11.31 (LOD  = 2.39; p = 0.0004) for muscle-mass adjusted isometric knee extension torque.

**Conclusions:**

We conclude that many small contributing genes rather than a few important genes are involved in causing variation in different underlying phenotypes of muscle strength. Furthermore, some overlap in promising genomic regions were identified among different strength phenotypes.

From a general health perspective, muscular fitness is associated with independently performing activities of daily living.^1^ Indicators of functional status of skeletal muscle (strength, power and endurance) are positively associated with bone health^2^ and psychological wellbeing,^3^ and negatively associated with falls and fractures,^4^ morbidity^5^ and mortality.^6^ ^7^ The age-associated decline in muscular strength and mass may also be related to prolonged disuse and/or chronic disease, such that a vicious cycle is created wherein inactivity leads to sarcopenia, which further worsens the ability to perform activities of daily living. Interindividual variability in muscular fitness and muscle mass raises the question of which genes, in addition to environmental factors such as nutrition, social status, and training, influence musculoskeletal fitness components that are determining factors for predicting health status, particularly in the elderly.

Several studies suggest that muscle and bone cross-sectional area (MBA) and isometric (F_isom_), concentric (F_conc_), and eccentric (F_ecc_) muscle strength are under moderate to high genetic control, with heritability rates of 60–95% for MBA, 44–78% for F_isom_, 31–61% for F_conc_, and 65–77% for F_ecc_.^8^^–^14 However, muscle strength is a complex multifactorial trait, and high heritability does not guarantee the presence of quantitative trait loci (QTL) with large effect size. Heritability estimates for variation in isometric muscle strength at various elbow angles, indicative of torque–length specificity, have been reported by Thomis *et al*.^12^ The genetic determination of maximum static strength was highest (78%) at the middle angle (110°), and decreased gradually over the flanking angles (75% at 140° and 66% at 80°) to the extreme angles (70% at 170° and 0% at 50° flexion).

The human gene map for performance and health-related fitness phenotypes^15^ indicates that only a few studies have reported significant association of allelic variants in putative QTLs with muscle strength characteristics, and even fewer associations have consistently been replicated. The linkage studies of Huygens *et al*^16^ ^17^ were the first to explore the role of candidate genes in the myostatin pathway, examining QTLs for knee or trunk muscle strength and estimated muscle cross-sectional area in 329 young Caucasian male siblings from the Leuven Genes for Muscular Strength study (LGfMS). Results from these single-point linkage explorations with a single microsatellite marker per candidate gene revealed that the chromosomal regions harbouring myostatin (*GDF8*; 2q32.2), p21 (*CDKN1A*; 6p21.2), MyoD (*MYOD1*; 11p15.1) and retinoblastoma (*RB1*; 13q14.2) are potentially interesting regions for further genetic studies (LOD scores between 1.50 and 2.78, p values between 0.05 and 0.0002). Strengthened by these preliminary single-point linkage results and the physiological evidence for the entire myostatin pathway, a multipoint linkage analysis was performed on a larger and partially independent sample^18^ for these genetic regions, in addition to the regions harbouring the muscle regulatory factors Myf5 (*MYF5*; 12q21.31) and Myf6 (*MYF6*; 12q21.31), insulin-like growth factor-1 (*IGF1*; 12q22q23), cyclin-dependent kinase-2 (*CDK2*; 12q13), and titin (*TTN*; 2q31.2).^18^ Significant or suggestive linkage with knee muscle strength was found for the regions comprising *CDK2* (LOD 3.4, p = 0.0004), *RB1* (LOD 2.74, p = 0.0002) and *IGF1* (LOD 2.6, p = 0.0002).

To date, only 5% of the total genome has been scanned for linkage with muscle strength characteristics. We therefore performed a genome-wide linkage scan using 6008 SNP markers, aiming to identify genomic regions harbouring candidate genes that cause variation in MBA, isometric torque and the torque–length relationship of the knee flexors and extensors.

We identified several promising chromosomal regions harbouring a set of candidate genes, with some overlapping regions for different strength characteristics, suggesting pleiotropic gene action.

## METHODS

The procedures used in this study were approved by the medical and ethical committee of the Katholieke Universiteit Leuven. Before participation, the purpose and procedures of the study were explained in detail and the subjects gave their written informed consent.

### Subjects

From the total sample of the LGfMS, 283 male siblings aged 17–36 years in 105 families were selected; this group was composed of 13 quads, 47 trios and 45 pairs of brothers, resulting in 309 pairwise comparisons. The sibling pairs were selected based on their level of discordance for different strength phenotypes. The recruitment procedures and subject characteristics have been described in detail previously.^16^ ^18^

### Measurements

A detailed overview of the anthropometrical and muscle strength measurements in the LGfMS project can be found in Huygens *et al*.^16^ ^18^

#### Body composition

MBA of the mid thigh was estimated based on the circumference of the mid thigh corrected for skinfold thickness at the mid thigh:

MBA  =  (circumference mid-thigh−(skinfold thickness mid thigh×π/10)^2^)/(4×π).

Measurements were taken by experienced anthropometrists and are described in more detail elsewhere.^19^

#### Muscle strength

The Cybex NORM isokinetic dynamometer (Lumex, Ronkonkoma, New York, USA) was used to assess the maximum isometric and torque–length knee strength characteristics. After a 5–10 minute warm-up period on an ergometer cycle and light stretching exercises, subjects were positioned on the dynamometer, following the manufacturer’s instructions. Anatomical zero was set at full extension of the knee and the rotation axis of the joint was aligned with the mechanical axis of the dynamometer. Two isometric and four concentric submaximal trials preceded the actual tests, to allow familiarisation with the testing procedure (angle, range of motion or velocity). Maximal isometric knee strength was measured at two angles (30° and 60°). At each angle, highest torque values (Nm) during a 6-second isometric contraction of three maximum flexion and extension contractions were retained for further analysis. Between each contraction, subjects rested for 30 seconds. In accordance with the torque–length relationship of a muscle, optimum strength is generated at longer muscle length—that is, at an angle of 60° for knee extension (quadriceps) and 30° for knee flexion (hamstrings). The torque–length relationship for knee flexion and extension was quantified by calculating the ratio of torque at the joint angle in which the lowest mean isometric force is produced (60° for flexion, 30° for extension) over torque at the joint angle with the highest mean isometric force output (30° for flexion, 60° for extension), multiplied by 100:

(isometric flexion torque at 60°/isometric torque at 30°)×100.

#### Baecke Sport Index

The Baecke Physical Activity Questionnaire^20^ was used to assess the level of daily physical activity and more specifically, subjects’ sports participation in the Sport Index. Descriptive statistics and phenotypical correlations between the different strength characteristics and covariates were calculated using SAS V.9.1.(SAS Institute Inc., Cary, North Carolina, USA).

### DNA collection

DNA was extracted using the Chemagic DNA blood kit on an automated Chemagic Magnetic Separation Module I (Chemagen, Baesweiler, Germany) and a Multiprobe I (PerkinElmer, Waltham, Massachusetts, USA) robotic station. An Oragene saliva kit (Genotek, Ottawa, Ontario, Canada)  was provided to siblings who were not able to deliver a blood sample. DNA from these saliva kits was extracted following the guidelines of the manufacturer.

### Genotyping

The Illumina SNP-based Linkage Panel IVb was used for genotyping. The panel includes 6008 SNP markers distributed evenly across the genome. The average and median intervals between markers were 482 kb (0.64 cM) and 298 kb (0.35 cM), respectively. The largest interval between successfully genotyped markers was 5.02 cM on chromosome 8. The Illumina markers were typed with the Illumina Beadstation 500GX, in accordance with the manufacturer’s standard recommendations. The genotype success rate was 99.6% (33 SNPs excluded due to low signal or cluster overlap). Only autosomal SNPs were included for further analyses.

### Statistical analysis

#### Heritability estimations

Upper-limit heritability of the traits (h^2^) were estimated using the variance-components (VC) analysis procedure in QTDT.^21^ This estimate includes common environmental variation in addition to the additive genetic component, because these factors can not be separated with sibling pairs only. Hence, it is called the upper-limit heritability.

#### Power analysis

The power to detect regions harbouring susceptibility loci was estimated using the Genetic Power Calculator^22^ based on the heritability rates estimated in QTDT (assumptions: θ = 0, additive QTL variance 30%, common residual shared variance 40%, no dominance, residual nonshared variance 30%, α = 0.05).

#### Linkage analysis

Nonparametric multipoint linkage analyses were performed on 22 autosomes using the revised Haseman–Elston regression algorithm (MERLIN Regress) outlined by Sham *et al,*^23^ implemented using MERLIN software V.1.1.alpha.^24^ This method requires specification of trait heritability, population means and variances, which were calculated using the Pedstats^25^ and QTDT programs.^21^ Before statistical analyses, all genotypes were checked for mendelian inheritance, using the error detection protocol in MERLIN.^24^ Deviations from Hardy–Weinberg equilibrium were detected using the PEDSTATS program.^25^ Polymorphisms with Hardy-Weinberg p values <0.001 were removed from further analyses. 

Simulated data for 100 genome scans were generated using MERLIN^24^ (under the assumption of no susceptibility loci) to estimate the significant and suggestive threshold for linkage.^26^^–^28 In each simulation, we retained the original pedigree structure and generated a new dataset with the same allele frequencies, marker spacing, phenotypes and any missing genotypes or data pattern. Any evidence for linkage in these simulated data is due to chance. The cut-off for suggestive linkage (LOD  = 2.23) was calculated as the mean of the genome-wide maximum LOD score from each genome scan, which determines the maximum peak size expected once per genome scan by chance alone. The significant linkage threshold (LOD  = 3.25) was defined as the maximum LOD score occurring with probability 0.05 in a genome scan (ie, 5 peaks of equal or greater size observed in the 100 simulations).

It is now a widely accepted practice to obtain empirical p values for reported LOD scores.^29^^–^31 Empirical p values were calculated through the use of 1000, 10 000 or 100 000 gene-dropping simulations  (related to the original p values of SNPs with LOD >1.5)  using MERLIN,^24^ under the assumption of no susceptibility loci. The empirical significance level of a LOD peak was then determined by counting the proportion of simulated (unlinked) LOD scores greater than or equal to the original LOD score. The linkage analysis was then repeated including muscle cross-sectional area of the mid thigh and additionally the Sport Index score as covariates. Results of the unadjusted >1.5 LOD regions were compared to the adjusted analyses, and newly identified regions in the adjusted analyses were added.

#### Combined linkage and association analysis

Tests for joint linkage and association in regions with suggestive and significant evidence for linkage were carried out using a quantitative transmission disequilibrium test (QTDT) with MBA as a covariate.^21^ Single-nucleotide polymorphisms (SNPs) in a −1 LOD region around SNPs showing suggestive or significant evidence for linkage in the unadjusted MERLIN regression linkage analysis were investigated. Fulker *et al*^32^ developed a method for simultaneous modelling of association and linkage for quantitative traits using sibling pair data that also controls for population stratification. Combined linkage and association analysis is a powerful tool for pinpointing functional loci responsible for a linkage signal. Assuming there is suggestive/significant linkage before modelling association, the extent to which evidence for linkage diminishes in the joint test of linkage and association reflects the proximity of the marker to the functional QTL.^32^ ^33^ If the linkage signal of a certain locus is entirely explained by modelling association (ie, the linkage signal is no longer significant), that particular locus is either the functional locus or in tight linkage disequilibrium (LD) with the functional locus.

#### Association analysis

SNPs in a −1 LOD region around the SNP showing significant evidence for linkage and SNPs in overlapping chromosomal regions between phenotypes were tested for association with the MERLIN likelihood-ratio test.^24^ Analysis of covariance analysis was performed (SAS V. 9.1) to confirm the association results in MERLIN, comparing the three genotype groups, using a “sandwich” option to account for dependency between scores within families. Additionally, carrier versus non-carrier groups for each allele were tested in two-group comparisons. Association tests in MERLIN and SAS used MBA as covariate.

## RESULTS

### Descriptive statistics

Table 1 shows the main somatic characteristics and muscle strength statistics of the 283 siblings, with corresponding upper-limit heritability rates (h^2^) estimated on the total LGfMS sample. Subjects had normal weight and height; the mean (SD) body mass index (BMI) of 22.9 (2.9) kg/m^2^ indicates that they were, on average, rather lean.

**Table 1:** Somatic characteristics and muscle strength statistics

Trait	Mean (SD)	h^2^*, %
Age	25.1 (4.5)	
Weight, kg	74.5 (10.4)	85
Stature, cm	180.2 (6.5)	92
Fat-free mass, kg	63.4 (6.5)	90
Body mass index, kg/m^2^	22.9 (2.9)	81
MBA mid thigh, cm^2^	171.8 (22.7)	85
Isometric torque at 30° flexion, Nm	147.9 (28.9)	90
Isometric torque at 60° extension, Nm	260.9 (53.5)	78
Torque–length flexion, %	81.2 (8.7)	37
Torque–length extension, %	61.4 (8.5)	38
Sport index	3.0 (0.7)	50

*Estimated on the total Leuven Genes for Muscular Strength sample (n = 748).

Torque–length relationship for knee flexion and extension was quantified by calculating the ratio of torque at the joint angle in which the lowest force is produced (60° for flexion; 30° for extension) over torque at the joint angle with the highest force output (30° for flexion; 60° for extension), multiplied by 100.

As expected, the upper-limit heritability estimate of stature was high (92%), and genetic determination of body mass and BMI were somewhat lower (85% and 81%, respectively). Fat-free mass and MBA mid thigh showed h^2^ estimates of 90% and 85%, respectively. In this sample, isometric torque at 30° flexion and 60° extension were under strong genetic control (90% and 78%, respectively), whereas genetic control of the torque–length relationships for flexion and extension was only moderate (37% and 38%, respectively). At most, only 50% of variability in the Sport Index could be accounted for by genetic factors.

Correlations between the different strength phenotypes ranged from −0.26 to 0.72 (table 2). Moderate to high correlations were found between MBA mid thigh, isometric torque at 30° flexion and isometric torque at 60° extension (r ranged between 0.48 and 0.72). All other correlations were low (r between −0.26 and 0.19), indicating the specificity of the torque–length characteristics of knee extensor muscles and flexor muscles.

**Table 2:** Phenotypic correlations between strength and torque–length characteristics and covariates

		MBA mid thigh	Baecke Sport Index	Isometric torque at 30° flexion	Isometric torque at 60° extension	Torque–length flexion
Baecke Sport Index		0.19				
p Value		0.0014				
Isometric torque at 30° flexion		0.48	0.12			
p Value		<0.0001	0.05			
Isometric torque at 60° extension		0.52	0.11	0.72		
p Value		<0.0001	0.07	<0.0001		
Torque–length flexion		−0.06	−0.005	−0.26	−0.22	
p Value		0.29	0.93	<0.0001	0.0003	
Torque–length extension		0.12	0.01	0.04	−0.12	−0.09
p Value		0.04	0.84	0.52	0.04	0.14

p Values calculated with Pearson test.

### Linkage analyses

**Figure 1:**
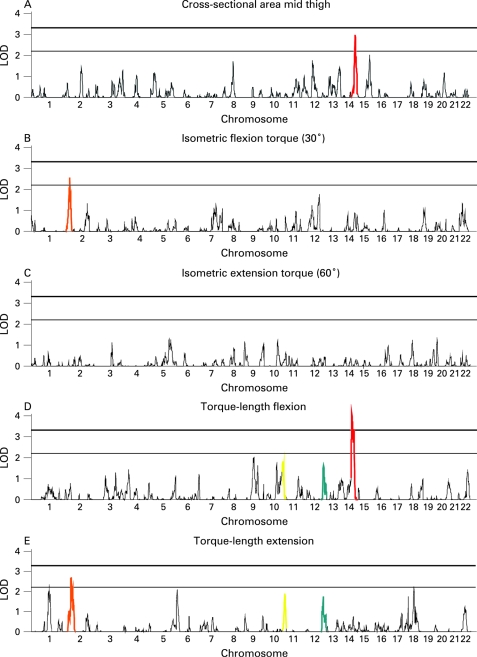
LOD scores for autosomal linkage analysis for muscle and bone cross-sectional area of the mid thigh and knee strength phenotypes. Cut-off LOD scores for suggestive and significant linkage are 2.2 and 3.3, respectively. Colours indicate overlapping or closely neighbouring chromosomal regions of interest between phenotypes.

Figure 1 shows LOD score curves over the autosomal genome for each of the unadjusted phenotypes. Empirical p values and LOD scores of ⩾1.5 are shown in table 3 (top panel). The highest multipoint LOD score (4.09) was found on chromosome 14q24.3 for the torque–length relationship of the knee flexors, with an empirical p value <10^−5^. Suggestive evidence for linkage (LOD between 2.2 and 3.3) was found for MBA mid thigh on 14q32.2 (LOD  = 3.00; p = 0.005), isometric knee flexion at 30° (LOD  = 2.57 on 2p24.2; p value  = 0.009) and the torque–length relationship of the knee extensors (LOD  = 2.33, 2.69 and 2.21 on 1q21.3, 2p23.3 and 18q11.2, respectively; p values <10^−4^ for all). No suggestive evidence for linkage was found for isometric knee extension at 60°. Overlapping or closely neighbouring chromosomal regions of interest were observed for torque–length flexion and extension (10q26 and 12q24), and for isometric torque flexion and torque–length extension (2p23-24) (as indicated by similar colouring pattern in fig 1). In general, adjustment of maximum isometric torques (at 30° flexion) for MBA lowered LOD scores substantially, whereas adjustment had only limited effects for torque–length phenotypes, except for increased LOD signals at 9q21.32 and 10q26.13 and a decreased LOD score for 14q24.3 for torque–length flexion. Additional adjustment for sports participation by the Sport Index score did not change LOD scores substantially. For MBA, correction for Sport Index scores resulted in lower LOD scores at 8q13.2 and 15q32.2, although other LOD scores remained equal. In the lower panel of table 3, chromosomal regions that did not meet the >1.5 LOD criterion in the unadjusted analyses, but showed regions with linkage >1.5 LOD after adjustment, were included. One suggestive LOD score was found for knee extension torque (at 60°) at 18p11.31 (LOD  = 2.394, p = 0.0004).

**Table 3:** LOD scores >1.5 and p values of multipoint linkage analysis for muscle and bone cross-sectional area (MBA) and knee strength phenotypes

Trait	SNP	Location	cM	LOD unadjusted	pValue	Empirical p value	LOD covariate MBA	LOD covariate MBA + Sport index
>1.5 LOD regions for unadjusted phenotypes (and comparison after covariation)								
MBA mid thigh	rs695167	8q13.2	79.78	1.719	0.002	0.016	—	1.254*
	rs296736	12q13.13	63.82	1.783	0.002	0.012	—	1.712*
	rs926949	14q32.2	109.13	2.998	0.0001	0.0049	—	2.854*
	rs7175643	15q32.2	99.82	2.048	0.0011	0.0102	—	1.078*
Torque at 30° flexion	rs1445128	2p24.2	41.12	2.572	0.0003	0.0097	1.532	1.615
	rs246079	12q24.11	124.26	1.791	0.002	0.018	0.335	0.463
Torque–length flexion	rs7846911	9q21.32	81.01	2.004	0.0012	0.0003	2.858	2.824
	rs4962424	10q26.13	153.12	1.81	0.002	0.003	2.524	2.524
	rs225553	12q24.32	147.91	1.615	0.003	<0.001	1.261	1.259
	rs760267	14q24.3	76.68	4.088	0.00001	<0.00001	2.972	2.895
Torque–length extension	rs13320	1q21.3	145.69	2.326	0.0005	<0.0001	2.064	2.032
	rs714513	2p23.3	50.2	2.690	0.0002	<0.0001	2.654	2.627
	rs9328112	6p25.2	7.27	2.079	0.001	<0.001	2.009	2.004
	rs1998825	10q26.3	166.36	1.877	0.002	<0.001	1.733	1.725
	rs169631	12q24.31	141.2	1.751	0.002	<0.001	1.308	1.291
	rs3785513	17q25.3	137.3	1.746	0.002	<0.001	1.644	1.669
	rs1010800	18q11.2	43.66	2.205	0.0007	<0.0001	2.173	2.175
Additional >1.5 LOD regions after adjustment for covariates								
MBA mid thigh	rs1880542	2q14.2	130.66	1.438	—	—	—	1.592*
Torque at 30° flexion	rs2056865	7q31.2	122.77	1.140	—	—	1.522	1.516
	rs8035183	15q23	74.51	0.262	—	—	1.853	1.777
Torque at 60° extension	rs1556611	10q23.31	109.44	0.117	—	—	1.566	1.695
	rs1941487	18p11.31	21.53	0.383	—	—	2.394	2.321

MBA, muscle and bone cross-sectional area of the mid thigh.

*Sport Index.

### Combined linkage and association analyses

**Figure 2:**
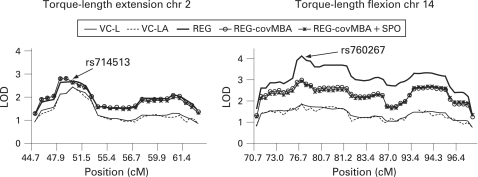
Representation of combined linkage and association analysis for torque–length extension and flexion at the LOD –1 support region around SNPs rs714513 and rs760267, respectively. LOD scores for variance components analyses in QTDT: linkage (VC_L) and combined linkage and association (VC_LA). LOD scores for MERLIN regression analyses: without covariates (REG); with MBA (REG_covMBA) and MBA+SPORTINDEX (REG_covMBA+SPO) as covariates.

Figure 2 shows two examples of LOD score curves for the effects of covariation on LOD scores and combined linkage and association analysis for SNPs in a LOD −1 support region around SNP rs714513 for the torque–length relationship of the knee extensors in regions 2p22.2–2p24.1 and for torque–length flexion at rs760267 (14q24.3). No significant drop in LOD scores was observed after modelling a joint test for linkage and association, indicating the absence of association between the investigated SNPs and the studied traits. Similar results were found for all other SNPs in regions with suggestive/significant evidence for linkage. However, when applying the MERLIN likelihood-ratio association test, significant association was found for two SNPs in close proximity of the highest linkage-peak SNPs: SNP rs935340 (14q24.3) was associated with the torque–length relationship of the knee flexors (p = 0.02) with decreasing effect of the A allele of 2.5 units. Classical AN(C)OVA-type association analysis confirmed the above MERLIN likelihood-ratio association findings. Carriers of the C allele for SNP rs935340 had higher torque–length values of the knee flexors than did the AA homozygotes (83.3 (1.2) vs 79.9 (0.8); p<0.01). SNP rs341173 at 18p11.31 was associated with isometric knee extensor strength (p = 0.018) with an increasing effect of the C allele of 11.2 units. AN(C)OVA association analysis did not confirm these results.

## DISCUSSION

We performed a genome-wide multipoint linkage analysis to identify quantitative trait loci for various characteristics of muscle strength, including MBA of the mid thigh, knee flexion and extension torque, and torque–length relationship for knee flexors and extensors. The most significant evidence of linkage was observed for torque–length relationship for the knee flexors with SNP rs760267 on chromosome 14q24.3. In this region, no evidence for linkage was found for any of the other measured strength phenotypes. It is possible, however, that the identified susceptibility locus is torque–length specific and does not account for variability in other strength or muscle mass phenotypes. As muscle strength and its derived phenotypes are regarded as a complex genetic trait, identification of a large number of markers with LOD scores that meet the criteria of suggestive linkage cannot be expected. In total, six chromosomal regions with a LOD score >2.2 were identified: 14q32.2 for muscle and bone cross-sectional area, 2p24.2 for knee peak torque at 30° flexion, 1q21.3, 2p23.3 and 18q11.2 for torque–length relationship of the knee extensors, and 18p11.31 for muscle-cross sectional area adjusted knee extension torque. These regions harbour several candidate genes for muscle strength phenotypes, although these regions were not reported in earlier linkage studies investigating strength-related phenotypes.

Our group previously performed a multipoint linkage analysis for muscle strength and muscle mass in a similar cohort of young male siblings, using microsatellite markers in chromosomal regions harbouring myostatin pathway genes.^18^ Significant or suggestive linkage was observed at chromosomal regions 12q14, 12q22q23, and 13q14q21, suggesting a role for *CDK2*, *IGF1* and *RB1* as possible QTLs for muscle strength.^18^ Apart from region 12q24.3, which was identified in the present study and is in the vicinity of the earlier reported region 12q22–23, no overlapping regions of linkage were found between the present study and the study of Huygens *et al*.^18^ Of note, SNP marker rs296736 with a LOD score of 1.783 (p = 0.012) for MBA is located at 12q13.13, which harbours the *CDK2* gene.

Compared with our previous analyses^18^ with microsatellites markers (average marker distance 4.81 cM for chromosome 12 and 2.32 cM for chromosome 13, respectively), the current study used the Illumina pre-designed SNP based Linkage IVb panel with an average marker distance of 0.56 cM for chromosome 12 and 0.49 cM for chromosome 13, respectively. Although this SNP panel induces higher genetic informativeness than microsatellite markers ,as shown by earlier reports,^34^ ^35^ differences between the present (n = 283; Illumina SNP panel) and previous findings (n = 367; microsatellite markers; Applied Biosystems, Foster City, California, USA)^18^ are probably related to sample size differences or region-specific informativeness of closely chosen microsatellite markers versus SNP markers (mean heterozygosity of SNP markers was 44.2% and 44.9% on chr12 and chr13, respectively).

The various examined phenotypes related to muscle strength are regulated by numerous genes, each contributing a small part to the total variability. In addition, gene–gene and gene–environment interactions and contributions of environmental factors for muscle strength can be assumed to affect the power of linkage studies that attempt to find single locus–trait relationships. The identification of susceptibility loci with a small sample size is therefore difficult. In addition, the power to detect linkage between a trait and genomic regions is further reduced in the absence of parental genotypes, owing to less accurate IBD estimations between siblings in the same nuclear family. Therefore, we selected for this study only the largest families from the total LGfMS sample. The sample included 13 quads, 47 trios and 45 pairs of brothers, resulting in 309 pairwise comparisons. With the current sample, the power to detect a QTL accounting for 30% of the phenotypic variance, 40% residual shared variance, no dominance, and residual non-shared variance of 30%, is estimated to be 85%. Although inclusion of measured environmental factors as covariates might increase power to detect linkage, inclusion of a measure of sports participation did not have a noticeable impact on LOD scores. In part, this is due to the low phenotypic correlation between the Sport Index and the strength measures, which might be attributed to the aerobic component that is used to assess the intensity of the activity. Furthermore, sports participation itself is not a true environmental component, as the Sport Index showed a considerable upper-limit heritability, which has been reported in several other studies.^36^ ^37^

Several multivariate genetic studies examined genetic and environmental contributions to individual differences in various muscle strength characteristics.^10^–^14^ ^38^ These studies suggest a shared pleiotropic gene action for the different phenotypic characteristics (the same genes causing variability for different types of contractions, speeds and angles), referring to a common underlying genetic cause of strength generality. This would imply that different examined phenotypes share the same chromosomal region with suggestive or significant evidence for linkage. Although no clear overlap for chromosomal regions between the different phenotypes was found, several regions (on chromosomes 2, 10 and 12) show some weak evidence for a common genetic strength factor that is shared between at least two phenotypes. In addition, by comparing unadjusted versus adjusted (for MBA) LOD scores, a lower LOD score for the adjusted phenotype might be expected if the chromosomal region is involved in MBA per se. This was found for the chromosome 14 region, where a drop in LOD from 4.088 to 2.972 was observed for torque–length flexion, close to a region where linkage for MBA was suggestive. However, other drops in LOD scores could not directly be linked to high LOD scores for MBA. Few additional regions were identified when strength phenotypes were adjusted for MBA. These might present muscle cross-sectional area -independent aspects in muscle contraction such as coordination and motor unit recruitment.

Previous studies have also suggested the presence of unique genetic causes of variance (genes causing non-shared variability in one specific muscle characteristic).^10^–^14^ ^38^ The observed differences in heritability for torques measured at different elbow angles was suggested to be related to differences in the importance of a subjects’ physiological muscle resting length and the moment arm at specific joint angles.^10^ Genetic components related to physiological rest length and general stiffness of muscles might be picked up in the analysis of the torque–length phenotypes in our study. The titin gene would be a good candidate for the active fraction of the muscular stiffness component within the sarcomeres, although its concrete role in human skeletal muscle stiffness is not yet fully understood,^39^ and lack of evidence of linkage was found in our study. In rat models, soleus fibres expressing the slow myosin heavy chain (MHC) isoform have been observed to be stiffer than fast extensor digitorum longus fibres with the fast MHC isoform.^40^ Transferred to humans, this would indicate that higher proportions of type I fibres would indicate higher stiffness of muscle. However, rabbit data show a less consistent picture of the relationships between titin length variability, MHC type and passive and active contractile properties.^41^ The passive fraction of the muscular stiffness, extra-sarcomeric intramuscular connective tissue, which is largely composed of collagen, might additively or interactively contribute to interindividual differences in the torque–length relationship of the muscle.^39^

Several candidate gene association studies for muscles strength phenotypes^42^ and a yearly update of candidate genes for health-related fitness phenotypes^15^ have been described. Reported candidate genes for muscle strength and muscle mass include *DIO1* (1p32p33), *GDF8* (2q32.2), *MYLK* (3q21), *NR3C1* (5q31), *TNF* (6p21.3), *CFTR* (7q31.2), *CNTFR* (9p13), *IGF2* (11p15.5), *CNTF* (11q12.2), *ACTN3* (11q13q14), *VDR* (12q13.11), *IGF1* (12q22q23), *COL1A1* (17q21.33) and *ACE* (17q23.3). Some of these genes are located in the vicinity of linkage regions reported in the present study: region 6p25.2 near to *TNF* (6p21.3), linked to torque–length for the knee flexors; regions 12q24.32 and 12q24.31 close to *IGF1* (12q22q23), linked with torque–length for the knee flexors and extensors, respectively; and region 17q25.3 near to *COL1A1* (17q21.33) and *ACE* (17q23.3), linked to torque–length for the knee extensors.

Apart from these “usual suspects”, other candidate genes can also be found in or near to the regions identified in our autosomal linkage analysis. For example, the hypoxia-inducible factor 1α gene (*HIF1A*), which has been reported to be associated with maximum oxygen consumption before and after exercise training,^43^ is located at chromosome 14q21q24, close to the highest LOD peak found in our study at 14q24.3 (for torque–length relationship of the knee flexors). However, although oxygen supply to the muscle is of critical importance to generate force and a limiting factor in endurance exercise, evidence for a direct link between variability in the torque–length relationship of the muscle and the amount of oxygen supply/consumption is currently unclear. Suggestive evidence for functional torque–length differences between runners and cyclists have been described by Herzog *et al*;^44^ however, differences between oxidative stress-related exercise adaptations were not reported in this study. Furthermore, the tripartite motif-containing 54 (*TRIM54*) gene (2p23.3) is a member of the muscle ring finger proteins, which are identified as myogenic regulators of the microtubule network of striated muscle cells and reveal a link between microtubule organisation and myogenesis.^45^ The ADAM metallopeptidase domain 12 (*ADAM12*) gene (10q26.3) has high expression in muscle development and regeneration, but its specific role in skeletal muscle is not yet understood.^46^^–^48 The protein kinase B gene *AKT1*, located at 14q32.32 (close to the suggestive linkage region for MBA at 14q32.2), codes for the Akt-1 protein, which has previously been associated with hypertrophy, and also has a key role in atrophy through the phosphorylation of a transcription factor, Foxo.[49, 50]  Further fine mapping analysis will be required to assess the potential contribution of each of these candidate genes to explain muscle strength variation.

Finally, results of the joint test of linkage and association showed no evidence for association with any of the SNPs in regions with suggestive or significant evidence for linkage. However, the association analysis using Merlin software^24^ revealed significant association with two individual SNPs: rs935340 in 14q24.3, and rs341173 in 18p11.31. The proportion of the variance explained by each of the SNPs is, however, low: 2.29% and 1.21%, respectively. These SNPs are located in the vicinity of the SNPs with highest LOD scores within their respective chromosomal region. Finding a “true” association between a trait and a SNP requires complete LD between the marker and the functional SNP, or the SNP being the actually functional SNP itself. Marker spacing in the Illumina Linkage IVb panel, designed to perform linkage rather than association analyses, is relatively large (average marker spacing 482 Kb or 0.64 cM), with low between-marker LD. Furthermore, the panel is not specifically designed to include functional SNPs (eg non-synonymous variants), as minor allele frequency and heterozygosity are the main inclusion criteria for markers. A much denser marker panel (eg microarray chips) or a functional fine mapping panel of SNPs located in relevant candidate genes will be needed to assess association in regions with suggestive or significant evidence for linkage.

## CONCLUSION

This genome-wide multipoint linkage analysis of various muscle strength characteristics revealed one chromosomal region on chromosome 14 with significant evidence for linkage with torque–length relationship of the knee flexors, and several other regions with suggestive evidence for linkage (for muscle and bone cross-sectional area, for isometric knee flexion torque and torque–length relationship of the knee extensors). Several candidate genes that might be relevant from a physiological point of view are located within the identified regions, and warrant further fine-mapping analyses by additional linkage and/or association studies. Although we selected the largest families from the LGfMS project, the relatively small sample size limits the possibility to detect chromosomal regions where genes with small effect sizes are harboured. Fine-mapping and replication studies of the current findings should therefore focus on highly informative samples (families) of sufficiently large size, and preferably extend to females and other age groups. The chromosomal regions identified within this healthy population might have implications beyond the field of muscular fitness and be directive in muscular disease research.

**Accession numbers**

The National Center for Biotechnology Information (NCBI) Entrez database accession numbers for the genes discussed in this paper are:

- Angiotensin 1 Converting Enzyme (*ACE*), 1277
- Actinin 3 (*ACTN3*), 89
- A disintegrin and metallopeptidase domain 12 (*ADAM12*), 8038
- Protein kinase B (*AKT1*), 207
- Cyclin-dependent kinase 2 (*CDK2*), 1017
- Cystic fibrosis transmembrane conductance regulator (*CFTR*), 1080
- Ciliary neurotrophic factor (*CNTF*), 1270
- Ciliary neurotrophic factor receptor (*CNTFR*), 1271
- Collagen type 1 alpha-1 (*COL1A1*), 403651
- Deiodinase iodothyronine type I (*DIO1*), 1733
- Myostatin (*GDF8*), 2660
- Hypoxia-inducible factor 1 alpha subunit (HIF1A), 3091
- Insulin-like growth factor 1 (*IGF1*), 3479
- Insulin-like growth factor 2 (*IGF2*), 3481
- Myosin light chain kinase (*MYLK*), 4638
- Nuclear Receptor Subfamily 3 Group C Member 1 (*NR3C1*), 2908
- Retinoblastoma (*RB1*), 5925
- Tumour necrosis factor (*TNF*), 7124
- Vitamin D receptor (*VDR*), 7421
